# Deliberative democracy and historical perspectives on American Indian/Alaska native political decision-making practices

**DOI:** 10.1057/s41599-020-0506-4

**Published:** 2020-06-24

**Authors:** Justin Reedy, Raymond Orr, Paul Spicer, Jessica W. Blanchard, Vanessa Y. Hiratsuka, Terry S. Ketchum, Bobby Saunkeah, Kyle Wark, R. Brian Woodbury

**Affiliations:** 1 University of Oklahoma, Norman, OK, USA; 2 Southcentral Foundation, Anchorage, AK, USA; 3 University of Alaska Anchorage, Anchorage, AK, USA; 4 East Central University, Ada, OK, USA; 5 Chickasaw Nation Department of Health, Ada, OK, USA

## Abstract

Public deliberation has risen to the forefront of governance as a technique for increasing participation in policy making. Scholars and practitioners have also noted the potential for deliberation to give greater influence to historically marginalized populations, such as Indigenous peoples. However, there has been less attention paid to the potential fit between the ideals of deliberation and the governance and decision making practices of American Indian and Alaska Native (AI/AN) peoples. In this paper, we begin to address this gap by analyzing accounts of AI/AN governance from the perspective of deliberation, and note areas of overlap, synergy, and conflict. We conduct a close reading of key historical and ethnographic accounts of four historical AI/AN contexts—the Iroquois Confederation under the Great Law of Peace, 19th century accounts of the Ojibwa village, the Santa Clara Pueblo government in pre-19th century, and Yup’ik village life in the early 20th century—and a more contemporary case in the form of the Santa Clara Pueblo’s Constitution from the Indian Reorganization Act period. We then apply two sets of key criteria for deliberative democracy—from the scholars Robert Dahl and John Gastil—to these accounts and note the ways in which each system is or is not congruent with these frameworks of deliberation. We find variations between these historical tribal contexts in our analysis. Social components of deliberation, such as respectful discussion and equal opportunities to participate, were partially or fully present in many accounts of governance practices, but it was less clear whether the analytic components, such as discussion of a range of solutions, were included in some forms of tribal governance. We then explore the potential implications of our findings for public deliberation within and in AI/AN tribes. We note that deliberative scholars and practitioners should be wary of over-generalizing about AI/AN tribes.

## Introduction

The rise of public deliberation, in both research and politics, inevitably raises questions about the origin and value of deliberative democracy, especially in the presence of competing political orders. While many have approached deliberative methods as tools potentially separable from their political and philosophical underpinnings, it is important to acknowledge their rootedness in this broader intellectual context, which shapes the methods in crucial ways. Such inquiry is especially indicated when we think about using these methods in the context of American Indian and Alaska Native (AI/AN) communities, where tribal entities regularly balance Indigenous and imposed forms of governance. The relationship between traditional tribal systems and democracy is of enduring interest, and both the structure of the U.S. federal government (Jacobs, 1991) and the centrality of liberty (Johansen, 1982) have been attributed to Indigenous, rather than European sources. Our goal in this paper is to examine historical accounts of governance and decision-making in AI/AN communities to understand the fit between these systems and contemporary articulations of public deliberation. Such an exercise is inevitably fraught with difficulties, since even at the time such accounts were written, AI/AN communities already had centuries of experience with European colonialism, but we suggest that it remains useful as a way of thinking through some of the challenges that can arise when thinking about the uses of deliberation in AI/AN communities. As Horn-Miller notes on the Haudenosaunee, a term used within the Iroquois to refer to ‘people of the longhouse’, ‘Community Decision Making Process itself is a bridge between old practices and the modern world’ (2013, p. 113).

Public deliberation has risen to the forefront in governance theory discourses in recent years both domestically (Gastil and Levine, 2005; Nabatchi et al., 2013) and internationally (Carson et al., 2013; Fishkin, 2009; Gastil and Levine, 2005; Souza, 2001), and questions of culture have increasingly been engaged (Abdel-Monem et al., 2010; Cronin and Ostergren, 2007). Exploring the connection of deliberation and tribal discourses is not without liabilities and we are wary of representing deliberation as either wholly consistent with or wholly opposed to AI/AN traditions. Such approaches fail to engage the complexities of both historical and contemporary AI/AN communities (Valadez, 2010; Sayre, 2000). In the past, when scholars used essentialism like “AI/AN peoples are more inclined to be ‘X’ than non-Native peoples” as a point of departure, the direction of the ensuing debate has not always been productive. Take, for instance, the longstanding debates over whether or not AI/AN peoples are or were more environmentally inclined compared to other ethnic groups (see Cronon, 1983; Kerch, 1999; Nadasdy, 2005; Orr and Ruppanner, 2016; Reo and Whyte, 2011; White, 1995). Other debates in scholarship include whether or not AI/AN peoples were more inclined to socialism (see Dunbar-Ortiz, 2015; Pinel, 2007) or political and social harmony (see Orr, 2017), or whether “sovereignty” was a concept appropriate for AI/AN peoples (see Alfred, 1999; Cobb, 2005; Barker, 2005). Debates about the role of deliberation in reinforcing tradition, solidifying stereotypes, or even expanding colonial authority have their place, but the purpose of this manuscript lies elsewhere. Rather, we present key components of public deliberation to explore how these might relate to historic and contemporary accounts of specific AI/AN governance practices.

Scholarly accounts and popular conceptions of AI/AN culture suggest a spirit of deliberation at the core of political processes found in many Indigenous North American communities—an interest in building consensus, for example, and the importance of talking through political conflict (see Horn-Miller, 2013 for a tribally specific example and Mohawk, 1978 for an international example). As deliberation has become increasingly salient in community governance scholarship and practice, including in AI/AN communities and in contexts involving AI/AN peoples (see, for example, von der Porten et al., 2015; Raymond-Yakoubian and Daniel, 2018), it is useful to consider how the principles of deliberation may reflect similar principles informing public dialog in AI/AN communities. At the same time, there may be important tensions between deliberation and some AI/AN community discussion practices. Additionally, the diversity of governance practices in tribes means that we cannot speak of distinct AI/AN communities as a single group with an identical set of communication practices. Furthermore, the imposition of non-indigenous governance structures on many tribes through the Indian Reorganization Act (IRA) in the 1930s could further complicate the relationship between AI/AN governance practices and the ideals of deliberative democracy, much like how governance structures like the U.S. Constitution do not always encourage deliberation in practice (Dahl, 1989). A deeper analysis of the variety of AI/AN tribal governance practices may help us understand areas of synergy and conflict between historical conceptions of AI/AN public deliberation and notions of public deliberation, as well as the potential consequences to deliberation when transitioning away from older forms of governance. By doing this, we hope to illustrate the complexity of connecting deliberation with the variety of AI/AN tribal governance practices and what that might mean for community engagement within AI/AN contexts.

In this paper, we briefly define some key principles of public deliberation and apply them to several AI/AN contexts, including four descriptions of historical AI/AN political practices—the Iroquois Confederation under the Great Law of Peace, 19th century accounts of the Ojibwa village, the Santa Clara Pueblo government in pre-19th century, and Yup’ik village life in the early 20th century—as well as a more contemporary case in the form of the Santa Clara Pueblo’s Constitution (developed in the 1930s). We describe whether and how each of these examples relate to several criteria that are important for deliberation (as defined by two notable scholars of democracy, Dahl and Gastil), and note ways that contemporary definitions of deliberation can miss notable democratic ideals present in AI/AN governance. We also discuss the implications of our analysis for present-day efforts to implement deliberative approaches in AI/AN communities. Some important caveats are necessary at this point: First, for the reasons discussed above, we do not intend for these cases to represent all contemporary practices or AI/AN peoples, and certainly not a generalized notion of AI/AN governance, nor do we try to represent any particular aspect of the practices described here as either traditional or non-traditional. Second, we are not claiming to provide a definitive account of governance practices in the cases we present. Rather, we are interested in describing a sampling of the variety of AI/AN governance practices across place and time as allowed by admittedly limited historical and ethnographic accounts as a stimulus to critical reflection of the extension of models of public deliberation into new cultural and political settings.

## Deliberative theory and deliberation processes

To guide our analysis of AI/AN discussion practices, in this section we review relevant scholarship on deliberative democracy and provide an overview of two models of democratic decision making from the U.S.—one a more general theory of a democratic process advanced by political theorist Robert Dahl, and the other a more specific model of the practice of public deliberation developed by deliberative scholar John Gastil. In addition, we provide a brief overview of scholarship on deliberation and AI/AN peoples, and note opportunities for further conceptual development in the research and practice occurring in these areas.

Political deliberation has connections to numerous theories of democracy and governance, from those developed by classical philosophers like John Locke (1690) and John Stuart Mill (1859) to ideas advanced by more modern scholars like John Rawls (2005) and Jürgen Habermas (1996). Some of this work has taken a macro-level approach, focusing on how political systems may facilitate or inhibit deliberation in the public sphere (Tocqueville, 1835; Cohen, 1997; Goi, 2005; Habermas, 1996; Knobloch, 2011). Other scholars have investigated meso-level and micro-level factors, such as the roles of political practices, organizations, groups, and individuals doing the work of deliberation (Nabatchi et al., 2013; Carcasson and Sprain, 2016), which is the approach we have taken in this paper. This research has examined deliberation in both formal and informal settings—that is, how deliberation can be facilitated through formalized structures (often written) and practices (Carson et al., 2013; Fagotto and Fung, 2006; Gastil and Levine, 2005), as well as the deliberative nature of discussion and dialog practices that occur in less formalized and naturally occurring settings (Eveland et al., 2011; Jacobs et al., 2009; Marques and Maia, 2010; Reedy et al., 2016; Wojcieszak and Mutz, 2009).

Deliberative democracy has risen in prominence in recent years in the U.S. and around the world, both as an area of scholarly investigation (Cohen, 1997; Habermas, 1996; Gastil, 2004) and a method of public engagement (Nabatchi et al., 2013). Public managers, political leaders, and citizens are increasingly recognizing the value of involving citizens and community stake-holders, including historically marginalized populations, in public decision-making processes (Carson et al., 2013; Lukensmeyer, 2013). In many cases, that involvement is taking the form of deliberative public engagement (Fung and Wright, 2003; Gastil and Levine, 2005; Karpowitz and Raphael, 2014; Nabatchi et al., 2013). Deliberative processes can produce policy decisions that are better grounded in citizen interests and values (Carson and Hartz-Karp, 2005; Fishkin, 2009; Gastil, 2004; O’Doherty and Burgess, 2009; Ratner, 2005) and can positively influence elections and other larger-scale political processes (Carson et al., 2013; Gastil et al., 2018). In addition, deliberative public engagement can improve citizens’ trust in political processes (Knobloch and Gastil, 2015) and increase the legitimacy of decisions (Richards and Gastil, 2015).

As deliberation has become a more common form of public engagement, it has taken on many varied forms, from small gatherings like citizen juries (Knobloch et al., 2013) to larger groups like citizen assemblies (Ratner, 2005) and deliberative polls (Fishkin, 2009), and from brief gatherings lasting a day or less (Lukensmeyer, 2013; Ryfe, 2006) to extended events lasting several days or even longer (Carson et al., 2013; Daniels and Walker, 2001). However, many of the underlying tenets of deliberation are shared across these different formats. In the following section, we describe two notable theoretical frameworks that guide deliberative engagement.

## Two frameworks for understanding deliberation: Dahl and Gastil

One notable political theorist, Robert Dahl (1989), has advanced a model for democratic processes that contains key concepts that inform many models of public deliberation. For our analysis below, we consolidate Dahl’s criteria into three key areas: *inclusion, opportunities for participation*, and *enlightened understanding* (see also, Gastil, 2008). First, Dahl’s model is grounded in *inclusion*: a democratic process should include essentially all adults within the body politic, Dahl argues. For a process to be considered sufficiently inclusive, it must allow for the participation of every adult governed by the decisions made within that democratic process, except for those with severe mental disability.

Democratic processes should also give citizens effective and sufficient *opportunities to participate* in the process. For Dahl, these opportunities must extend beyond the ability to vote to include opportunities to debate and discuss issues under consideration. Although Dahl’s model is less focused on discussion than is Benjamin Barber’s conception of democracy (1984), it does emphasize the importance of enabling citizens to express their views and question those of others. Dahl also argues that citizens should be allowed to shape the political agenda by bringing important questions up for consideration, rather than the agenda simply being driven by political elites. In addition, citizens should be given the opportunity to vote on those issues with equality of voting power at the decisive stage of decision making. Dahl argues that preceding stages could privilege some voices to ensure their equal consideration overall—that is, underrepresented or marginalized citizens could be given greater weight at certain stages, as long as equal voting power is retained at the decisive stage.

The third major criterion in Dahl’s model is *enlightened understanding*. Under this criterion, processes should allow people to get adequate information about issues and prospective choices, and about the potential consequences of different policy outcomes for themselves and others. People should also be able to connect those choices to their underlying values and conceptions of the public good. Dahl is intentionally vague in describing how exactly this should work, to allow for different ways for citizens to learn about and analyze political issues. The implication of his criterion is that citizens would be well served to engage in thoughtful reflection and analysis of policy choices, perhaps guided by discussion with their fellow citizens and other forms of information gathering, which is strongly aligned with the ideals of public deliberation.

There are more specific theoretical models than Dahl’s for public deliberation. One such framework advanced by John Gastil (2008) identifies the *analytic components of deliberation*, which focus on information about issues and relevant analysis of them, and *the social components of deliberation*, which are concerned with the behavior of citizens and interactions between them as they deliberate. Gastil defines deliberation as a process of an inclusive gathering of people engaged in thoughtful discussion and analysis of an issue, often guided by a moderator or facilitator, resulting in a well-reasoned decision or set of shared findings (Burkhalter et al., 2002; Gastil, 2008). Gastil’s framework for deliberation is supported by other scholarship that has identified several communicative and cognitive behaviors that help make up a deliberative process of discussion and decision making (Cohen, 1997; Mansbridge et al., 2006).

In Gastil’s framework, the analytic process focuses on how deliberative participants understand information and arguments during a gathering. The analytic components under this framework include building a strong base of information; identifying the key values at stake; discussing a range of potential solutions; weighing the pros, cons, and tradeoffs of those solutions; and applying one’s values and knowledge in making a decision. All four of these components are in keeping with the deliberative ideals of having a process with a thorough analysis of information and viewpoints (Burkhalter et al., 2002). Much like Dahl’s framework above, Gastil’s model notes the importance of people fully understanding their prospective policy choices through gathering and analyzing information, and by connecting that information or knowledge to their underlying values and conceptions of public good.

Equally important under Gastil’s framework are the social components of deliberation. These components emphasize the key social qualities that should guide a deliberative gathering. These components include respect for other participants at the gathering, equal opportunity for all to participate during the discussion, ensuring mutual comprehension among all participants, and the consideration of others’ ideas and experiences. Ensuring mutual comprehension and encouraging consideration of others’ ideas and experiences are grounded in the deliberative ideal of establishing a dialog that helps bridge gaps between citizens with sometimes disparate views (Burkhalter et al., 2002). Respect for other participants and equal opportunity to participate are important for establishing an egalitarian process of interaction between citizens, a key aspect of deliberation as noted by Cohen (1997). These social process components are also analogous to some aspects of Dahl’s model, such as the inclusion of all citizen voices, equality of participation opportunity, and thoughtful consideration of others’ perspectives.

It is important to note that although the frameworks from Dahl and Gastil place some importance on a group or society reaching a decision, not all conceptions of public deliberation are oriented toward such outcomes. For instance, some deliberative processes are aimed at highlighting areas of agreement shared by at least some of the participants as well as important areas of disagreement; those points of agreement and discord become the output of a deliberative forum and may be used to guide further discussion and policy making (O’Doherty and Burgess, 2009). Deliberation scholars have also argued that deliberative processes can be viewed as part of a cycle of inquiry and problem solving within a particular community, rather than a linear process in which a forum is created and decides before being disbanded (Carcasson and Sprain, 2016). In this model, deliberative forums help a community analyze potential solutions and also identify tensions between competing values and conflicting priorities. Communities are then able to use these findings to develop strategies for collaborative action, which may in turn lead to additional deliberative forums to refine and implement those strategies (Carcasson and Sprain, 2016). Still other scholars and practitioners in the realm of public engagement have noted the value of processes of dialog, which are often oriented toward building understanding between participants, rather than producing joint decisions and shared findings (Britt and Smithberger, 2018; Carson, 2011; Cramer and Walsh, 2007; Davidson and Moses, 2012; Judkins, 2012).

## Work on deliberation in AI/AN populations

Understanding deliberation in minority political and ethnic groups is not without precedent. Though some studies have looked at the challenges that ethnic and cultural heterogeneity posed to deliberative practices (see Mendelberg and Oleske, 2000; Makariev, 2013 or De La Fuente, 2013 for instance), other studies have looked at ethnic, cultural, and religious minority populations’ deliberative practices. Molina-Markham (2014), for instance, looked at Quaker meeting groups and their notion of interaction. Lundmark and Matti (2015) used the management of wildlife to see the effects of Saami (Indigenous Scandinavians) deliberative practices on ecological outcomes. Wheatley (2003) explores the appeal of deliberative decision making for ethnically and culturally marginalized groups as a means of integrating their political voice into representative systems. Rockloff and Lockie (2006) found that deliberative approaches to decision making among Indigenous Australians over shared natural resources with non-Indigenous populations was a promising model but required a specific emphasis on collective capacity.

Research involving AI/AN populations is of growing interest in the field of deliberation. This comes from perhaps a longstanding sense that AI/AN peoples’ governance is grounded in clan or tribal groups and, therefore, local (see Valadez, 2010 or Horn-Miller, 2013 on AI/AN community decision-making). Certainly, worIvison,ks in anthropology have focused on how AI/AN peoples seemed to be inclined to discuss decisions of importance to the group at a local scale (see Lansing, 2007 for coordination and Horn-Miller, 2013 for appropriateness). Though local, AI/AN governance could also be quite complex and akin to something larger, such as the Iroquois Confederacy. Both Benjamin Franklin and Thomas Jefferson, among others, noted the ability of the Iroquois to link village level interests to large scale collectives (see Jacobs, 1991 for a discussion of “Founding Fathers” interest in American Indian governance).

In addition to literature on deliberation that is internal to Indigenous governance is a larger literature on AI/AN peoples’ inclusion in deliberation centered around non-Indigenous populations (e.g. Carson et al., 2013). Ivison (2010) explores the use of deliberative democracy and reconciliation between Indigenous and non-Indigenous populations. Whereas most deliberative forums focus on appropriate action for the future, Ivison explores the use of such forums for retrospective approaches to past injustices and argues such forums may offer greater legitimacy and stability from the perspective of minorities. MacGinty (2008) has looked at AI/AN notions of creating peace in contrast with Western notions. Valadez (2010) considers deliberation as a way to manage post-violence negotiations between Indigenous Mexicans and government around resource use. In the Canadian context, von der Porten and de Loë (2013) look at how Indigenous peoples might be brought into collaborative agreements over water resources. Latimore et al. (2017) discuss the use of deliberation to control Indigenous public spheres among Indigenous Australians. A study that has not been carried out is to test the presence of deliberative practices across several Indigenous peoples and examine cross-cultural variation. As deliberative forms of public engagement have proven beneficial for many communities and for marginalized peoples, it bears asking how deliberative principles and Indigenous governance practices may connect and conflict.

## Cases

To develop a better understanding of how AI/AN communities may have engaged in deliberative democratic engagements, we selected governance systems in four tribal contexts and then look at a more generic tribal constitution from the IRA in the 1930s that many AI/AN tribes use today. These tribes and governance systems are the Iroquois Confederacy’s founding document *Kayanerenkó:wa* (“The Great Law of Peace”) that dates to the 13th century, Yup’ik peoples’ governance practices according to modern accounts of early 20th century village life, Ojibwa peoples’ conception of village politics in the 19th century, and both an account of the Pueblo of Santa Clara’s traditional religious governance system and its IRA-era constitution. We selected these tribal governance systems as examples in lieu of proposing to conceptualize the entire “Indigenous experience” or a notion of “Indigenous governance,” which is too large and varied to make claims to regarding its deliberative nature in an entirety. This sample is non-representative (though it does vary by region, context, and time period) and we have selected published accounts for sources on these governing systems, with a focus on original texts and on authors who rely directly on indigenous sources or are indigenous themselves. Given the qualitative nature of this study that relies upon five cases, we rely upon a medium-number (N) approach outlined by Rihoux and Ragin (2003). Though most social science research utilizes either small-N qualitative studies to gather a greater understanding of depth and nuance, or large-N quantitative studies to chart patterns and associations, our aims lie in appreciating the scope of variation possible in recorded deliberative practices. Given our aims and the fact that our “data,” in the form of accounts of AI/AN deliberation, do not lend themselves to “coding” in a numerical sense, this study fits within the case study method and comparative method as outlined by Lijphart (1971). We are not trying to exclude rival explications as we are not offering an explanatory claim as to the causes or effects of deliberative practices in AI/AN that a large-N study would offer. Nor are we attempting to generate a hypothesis that small-N studies would offer. Rather, we are following similar works in Native studies (see Cornell and Gil-Swedberg, 1995; Lerma, 2012) that use medium-*N* approaches to governance.

We hope by exploring five AI/AN governing systems, even a sample that is non-representative and not comprehensive or comprised of complete accounts, through the lens of deliberation will illustrate some of the variation found over time. What emerges in this analysis is (a) that tribal governance systems might differ from conceptions of deliberation as it is defined by western sources; (b) the epistemic doubt and difficulties involved in comparing tribal governing systems along the lines of deliberation; and (c) that a simple typology of deliberative versus non-deliberative practices may be insufficient as tribal governance systems we analyzed in some ways fit the criteria of deliberation by Dahl and Gastil and in some ways do not.

### Haudenosaunee (Iroquois) Confederacy and the Kayanerenkó: wa (“The Great Law of Peace”).

The Iroquois peoples or *Haudenosaunee* (which is typically translated as “People of the Longhouse” though other translations include “they are making a house” (Williams, 2018)) are a political, cultural, and linguistic group that mostly reside today in upstate New York and Canada. The political organization of the Haudenosaunee, also known as the Iroquois Confederacy, was originally comprised of the Cayuga, Seneca, Mohawk, Onondaga, and Oneida tribes, with the late addition of the Tuscarora around 1722 (Morgan, 1901; Sturtevant et al., 1978). The Six Nations of the Iroquois Confederacy offered both a military alliance and system of governance, used originally to “resist the pressure of contiguous nations” (Morgan, 1901) and to strategically pursue collective political and economic interests. Thought to be established as early as the 15th century (CE), the Haudenosaunee Confederacy was codified by wampum in the Great Law of Peace (Kayanerenkó:wa) and included 177 articles. As a governance system, its design was to reduce conflict and increase coordination among Haudenosaunee people and is considered to be a possible inspiration for the American Constitution (Jacobs, 1991).

The governance system created by the Great Law of Peace delegated responsibilities between representatives of tribes who were referred to as *hoyaneh*, which can be translated as “caretaker of the peace” or “counselor of the people” (Harris and Johnson, 2009). *Hoyaneh* from each of the tribes were assembled at a council and hosted by the Onondaga whose role were the “fire keepers”. These councils decided upon what policies could be taken by the Confederacy, though each nation maintained its own independence and autonomous interests; “the crowning feature of the League”, as stated in the enduring ethnographic accounts of Lewis Henry Morgan, “was the perfect independence and individuality of the national sovereignties, in the midst of a central and embracing government, which presented such a cemented exterior that its subdivisions would scarcely have been discovered in the general transactions of the League” (Morgan, 1901). No meeting was legal unless delegations from each tribe were present. Each convened council selected a speaker for the day from the Mohawk, Onondaga, or Seneca tribe (not the Oneida or Cayuga). Decisions were introduced into the council and need to be approved by *Hoyaneh* from each tribe, in consultation with their respective tribes. Within this system, women (clan mothers) were charged with selecting or removing of *Hoyaneh* during times of transition (Jacobs, 1991). The Confederacy coordinated the collective interests of the Iroquois until the Revolutionary War, when the political turbulence of the late 18th century prompted political divisions within the Confederacy causing each tribe to pursue their own interests. The Revolution contributed to the dispersal of the Confederacy across national borders, and it continued to take on new political roles and adopt new decision-making mechanisms relevant to differences in reservation life experienced by each tribe (Fowler, 2007; Tooker, 1978; Weaver, 1984) (See Simpson, 2014 for an ethnographic account of the Kahnawà:ke Mohawks’ historical and contemporary struggle to articulate and maintain political sovereignty or Crawford, 1994 for conceptualization of Iroquois Confederacy in an international relations framework).

### Yup’ik village historical governance.

The Yup’ik people are an Alaska Native population with a traditional homeland in southwestern Alaska, in a region several hundred miles west of Anchorage, where the Yukon and Kuskokwim rivers empty into the Bering Sea. Until the 20th century, Yup’ik life was centered in small family villages and nomadic camps in the lowlands of southwestern Alaska based around seasonal hunting, fishing, and foraging (Ayunerak et al., 2014). Though life for the Yup’ik people, or Yupiit, has changed in recent years, many in the community have expressed interest in preserving knowledge and understanding of the traditional Yup’ik way of life, and much of the population still lives in small communities in the Yukon–Kuskokwim Delta region.

Accounts of Yup’ik life in the recent past provided by community elders, Yup’ik scholars, and anthropologists help illustrate how the Yup’ik people govern village life and interactions with visitors from other villages and outsiders from non-Yup’ik cultures (Ayunerak et al., 2014; Fienup-Riordan, 1994, 2005, 2012; Hensel, 1996; Jacobson and Alaska Native Language Center, 1998; Napoleon et al., 1996). Villages were typically led by one person or a handful of people, generally village elders, who through a process of consensus and deliberation, made decisions of importance for the village, and who were seen as drivers and educators for the rest of the community. Leaders typically made initial decisions on their own, but decisions were finalized only after discussion with the community in the *qasgi* or *qasgiq*, the communal men’s house, and by presenting their plans and discussing with the other people in the village (Fienup-Riordan, 2005). Qasgiq is also a term that represents a process of collective discussion and action that persists in contemporary Yup’ik life, even as the physical structures of qasgiq may not (Rasmus et al., 2019).^1^

Social hierarchy played a role in determining who was involved in leading villages, but people who put in the effort to take part in community events and gatherings could be included in future decisions (Fienup-Riordan, 2005, p. 201), as were young people who were seen as particularly gifted or wise. Yup’ik villages were primarily led by men, but in some villages women elders were included in decisions as well. Unity was especially important in Yup’ik village life: community members were expected (though not forced) to go along with decisions once they were finalized, and in general, villages aimed to be harmonious and avoid excessive conflict (Fienup-Riordan, 2005; Rasmus et al., 2019). In general, Yup’ik society was “fundamentally relational rather than individualistic” (Fienup-Riordan, 2005, p. 55), which played an important role in village governance practices and the relative value of collective and individual interests.

### Santa Clara Pueblo governance (pre and post-IRA constitution).

Located in the central northern part of New Mexico, the Santa Clara Pueblo is part of the Pueblo cultural and Tewa linguistic group that are mostly clustered around the Rio Grande. Santa Clara, along with most Pueblos, were colonized first by the Spanish in the 16th century. Despite brutal subjection, most Pueblo tribes were not removed or relocated from their established villages, though the Spanish consolidated smaller villages into larger ones. The communal nature of the pueblo, as well as notions of their self-sufficiency (which was related to their lack of removal), later became inspirations for the re-emphasis of tribal politics in federal Indian policy (Kelly, 1983).

Pueblo governance has been the subject of scholarly study since the mid-20th century (Ortiz, 1969; also see Hill and Lange, 1982 for an ethnographic account of Santa Clara Pueblo political organization). Dozier (1966), who was himself a Tewa Indian from Santa Clara Pueblo, wrote about the hierarchical and religious nature of pueblo political order. Most pueblos have a *cacique* or civil leadership (compared to military leadership) who is decided upon by the leaders within the pueblo. In Santa Clara, before a political schism in the 1890s (Dozier, 1966; Hill and Lange, 1982), there were two cacique who traded control of the village affairs every 6 months. In 1935, tribes, including the Santa Clara, were pressured to incorporate written constitutions (often from a national template) as their foundation governing text. This constitution reorganized government in Santa Clara and developed a system that had an executive branch (Governor and Lieutenant Governor elected by adult tribal members) and legislative (eight Council Representatives elected by adult tribal members). The cacique still manage religious affairs for the tribe, but formalized political leadership was voted upon by adult tribal members.

### Ojibwa and 19th century accounts of village politics.

The Ojibwa peoples are part of the Anishinabek cultural group and Algonquin language family. Their traditional homeland was the Great Lakes region at the time of contact with Europeans and many still live in their traditional territories in the United States and Canada. Individual Ojibwa Nations, as defined by federal recognition, are located in Wisconsin or Minnesota, with the latter being those groups that were forced to move west in the mid-19th century under relocation policies. Older governance practices were based primarily at the village level (Miller, 2010). These older practices are outlined in both Ojibwe oral traditions, language and outsider accounts of political life in Ojibwe communities in the 19th century, and is knowledge that has been passed down in the communities themselves (Miller, 2010).

Accounts of Ojibwa political life comprised villages, which often had several hundred members, and a larger alliance system across villages. At the level of village, male *ogimaa* were the hereditary leaders and below them were the *gichi-anishinaabeg* (headmen) of each family who formed something like a council of village families. Outside of ritual or ceremony, neither the *ogimaa* or the *gichi-anishinaabeg* dressed differently than other Ojibwa (Miller, 2010, p. 77). Despite hierarchy in this system, accounts of village-level governance practices emphasized the degree to which consensus was necessary and generated through deliberation. “[O]gimaa seldom made decisions without consulting the gichi-anishinaabeg of each family lineage,” according the Miller (2010, p. 104). This seems to be a system of consultation that often frustrated outsiders as a fur trader noted of the Ojibwa “[they] rarely make their answers til the day after they have heard the arguments offered.” A consensus was expected within deliberations as the *ogimaa* had to be vigilant that he spoke for the agreed upon village when negotiating with outsiders. In this system, voices in tribal meetings were listened to by the *ogimaa*. We do not know what discretion the *ogimaa* had, but major decisions required defined roles and member consensus.

## Exploratory analysis

The results of our analysis of these historical accounts appears in the following section, and are summarized in Table 1.

### Iroquois case.

In the case of the Iroquois, the Great Law of Peace partially satisfied the criteria of inclusion and participation opportunities. *Hoyaneh*, whose powers were joint and co-extensive, were representatives of each nation within the confederacy. As part of a political class that had a more powerful political voice than other tribal members, *Hoyaneh* represented the great deliberative body of the League whose role was to be “counselor of the people” (Morgan, 1901). Discussions at the Great Council of the League were limited to men of elite status, and these individuals were carefully chosen through a process of localized decision making within individual tribes of the Confederacy. Though other men and all women were excluded from discussions themselves, they played roles in processes leading up to the Great Council discussions. The discussions held by the *Hoyaneh* were also consistent with the social components of deliberation that focus on respect and position. The social position of *Hoyaneh* also required them to consider how the potential actions by the Council affected their clan, tribe, and the larger Confederacy, which is very much in line with Gastil’s conception of the analytic components of deliberation. This broad mindedness was also temporal and required *Hoyaneh* to consider future generations— as far as seven ahead—in their deliberation (Graham, 2008).

### Yup’ik case.

For the Yup’ik people, accounts of village-level deliberation suggest it was inclusive, but discussions primarily involved male elders first, followed by discussions with other men in the village; however, women and particularly bright young people were included in some cases, and women sometimes served as village leaders. These discussions seemed to meet the criterion of participation opportunities: Leaders typically shared their thoughts with village residents on important decisions faced by the communities, and residents could comment but likely mostly listened. The Yup’ik worldview, which is strongly grounded in compassion and understanding for others, seems to be key to many community decisions; speakers supported their statements by citing the Yup’ik moral code, traditional narratives, and natural and spiritual signs (e.g., the disappearance of game animals), as well as statements about what they have personally witnessed. In addition, group discussions continued until the village was of one mind about a decision, suggesting that people were given adequate time to reflect on decisions. As noted above, the central location for deliberative discussions to take place was the *qasgi* or *qasgiq*, the men’s communal house, and an expectation there was for discussions to be respectful and to allow for the voicing of many different views, in keeping with the social components of deliberation described by Gastil. There were some described norms in the accounts of village discussion around analytic expectations in deliberations; decisions were to be carefully arrived upon, and as noted above these discussions relied upon a range of different kinds of evidence and support, including personal experience, the community’s moral code, spiritual beliefs, and traditional narratives.

### Santa Clara Pueblo (pre- and post-IRA) cases.

Accounts of pre-IRA governance practices in the Santa Clara Pueblo did not emphasize inclusion or participation opportunities, nor the social components of deliberation. There were no expectations that community members deliberate over community decisions openly. But leaders were supposed to maintain and work in accordance with Tewa cultural and religious values, which can be linked to the criteria of enlightened understanding and analytic components.

Accounts of governance outlined by the Santa Clara Pueblo’s IRA Constitution show mixed results regarding inclusion and participation opportunities. Under the IRA Constitution adult tribal members could vote and it is likely politics were discussed openly. The social and analytic components of deliberation are less clear in these accounts; the language of the IRA Constitution has no explicit requirements for discussion to occur in ways that are in line with Gastil’s criteria.

### Ojibwa case.

From accounts of 19th century Ojibwa village politics, young people were excluded from discussions around important decisions. Leaders were expected to call meetings when decisions affected the village, therefore there were opportunities for participation. Accounts suggest that leaders were expected to follow expectations for enlightened understanding towards the benefit of the group and instantiate analytic components of deliberation. At the inter-tribal level, leaders were required to maintain mutual respect but it is unclear that village level discussions had similar expectation or observed the social components of deliberation.

### Overall patterns.

Multiple themes emerge from the interaction of key components of notions of public deliberation and AI/AN governance practices. Variation along tribes is clear with some traditional governance practices resembling components of public deliberation more than others. For instance, the Iroquois Confederacy had multiple overlaps with aspects of public deliberation as outlined by Dahl and Gastil. The Iroquois Confederacy met criteria for two components and partially satisfied another two of the components key to public deliberation. Santa Clara Pueblo governance, as described by Norcini (2005), on the other hand did not have many attributes specified for public deliberation. None of the accounts we explored were fully in line with deliberation, nor did any of them show a complete absence of discussion components desired by deliberative theorists.

Most accounts of tribal governance practices included *participation opportunities*. Only the Santa Clara systems, both “traditional” and IRA Constitutional, showed a lack of the incorporation of participation opportunities as emphasized in public deliberative theory. Social components of deliberation were present in many of the accounts. This is in sharp contrast to the analytic components, with few accounts of traditional governance systems (or IRA) emphasizing this quality of public deliberation.

A sharp contrast between tribes appears on specific components. Yup’ik allow for the inclusion of some young people into discussions. In accounts of Ojibwa, young people are excluded from actively participating in deliberation. Whether the contract be on the overall presence of public deliberation components in historical accounts of tribal discussion practices (highly present in Iroquois and not Santa Clara Pueblo) or specific factors, such as the inclusion or exclusion of youth (Yup’ik in contrast to Ojibwa), the variation has important implications for thinking about who to include in a discussion process and why.

## Discussion and implications

It is clear that AI/AN governance varies considerably based on analysis of multiple deliberative criteria. Why governance practices vary is undoubtedly an important question but outside of the goals of this paper. However, this variation raises several implications for public deliberation in AI/AN communities. First and foremost, we should not hastily generalize about the ubiquity of deliberation as a component of tribal governance. Though aspects of this perspective might be true for certain groups such as the historical example of the Iroquois, it is not for others such as the account of traditional Santa Clara government. While most of these accounts emphasize limited inclusion and participation, all emphasize the priority of enlightened understanding through the need to consider decisions with respect to the communal values as established by the group. Interestingly, it is the IRA government that fails to meet most of the criteria for deliberative democracy, despite its ostensible origins in the democratic ideals of the U.S. As IRA-formed tribal governments use similar templates (Lemont, 2006), it is likely that shifts to this system at least decreased the formalized background of governing institutions away from rather than toward deliberation (see Biolsi, 1998). This shift away from deliberation in IRA constitutions fits with extant research on the movement away from older forms of governance that were more inclusive in other ways. For instance, Lerma (2014) found that adopting IRA constitutions were followed by a shift away from the inclusion of female tribal members toward a tribal politics that was more male. Whether greater male control of politics and the diminishment of older forms of deliberation are a part of the same feature is unknown but more work research should be undertaken on this relationship as lower political participation by women is associated with authoritarianism (see Fish, 2002).

We should be wary of oversimplifying the practices of AI/AN peoples, which has been a long-standing tradition in American popular culture and academic scholarship (see Sayre, 2000). What this analysis suggests is that it is inaccurate to equate AI/AN governance and traditional practices with modern Western practices of deliberation as described in this article. In the same way that extant research has found variation in tribes according to the appropriateness of concepts like sovereignty (Alfred, 1999), ecological stewardship (Krech, 1999), political and social harmony (Orr, 2017), or socialism (Barsh, 1988), the congruity of deliberation to AI/AN communities depends on the community and context. In some cases, as described by Horn-Miller (2013) and the current manuscript about the Iroquois Confederacy, deliberation as envisioned by Gastil and Dahl might work within traditional governance practices or at least merge with them. Therefore, there may be many points of synergy between deliberative principles as understood by Western scholars and AI/AN governance practices. In pursuing this line of research, we should be wary of the two traps of Western or non-native populations interacting with AI/AN communities, which are understanding principles of public deliberation as either a “natural fit” for, or a colonial expectation grafted onto Native communities.

Just as AI/AN governing practices are not uniform across multiple tribes, even individual tribes are heterogeneous and involve members with differing perspectives. Deliberation practitioners and researchers should be aware of the variation within as well as between tribes. For example, tribal members may vary on levels of interest in tribal politics, community engagement, and the degree to which their identity or sense of self is informed by tribal membership. Using demographic data, Nagel (1996) identifies national level variation of those who identify as AI/AN. Tribes are often diverse and comprised of multiple experiences and variation in that experience. Tribal members differ in adherence to contemporary or older political governance practices. When designing deliberative forms of engagement in tribal communities, practitioners may wish to consider different kinds of processes that can best address the social topography of diverse communities (see, for example, Strickland et al., 1999). For instance, some forms of deliberation may be better suited for dealing with groups dealing with long-standing conflict (see, for example, Fishkin, 2009; Ugarriza and Caluwaerts, 2014), whereas others could better address cultural variation or geographic separation within a community (Fagotto and Fung, 2006; Karpowitz et al., 2009; Lukensmeyer, 2013; Siu and Stanisevski, 2013).

In light of the growing interest in deliberation as a method of public engagement, one important contribution of this exploratory analysis is helping scholars and practitioners be aware of potential challenges of deliberation in AI/AN communities. This supports previous research by Young (2002) and Sanders (1997), for instance, that suggest that community dynamics and societal privilege must be taken into account in organizing systems of public discussion and decision making (also, see Chambers, 2003; Hickerson and Gastil, 2008; Walmsley, 2010). Neither scholars nor those facilitating deliberation with AI/AN communities can safely rely upon deliberation falling within AI/AN governance practices for all AI/AN communities or in the same way. By using Dahl and Gastil’s framework we also see that elements such as enlightened understanding and the analytic components of deliberation might have more emphasis than opportunities for participation and inclusion.

A major complication for implementation of deliberation is the degree to which a tribe or community, or the diverse group of individuals who comprise it, might be governed by different systems of governance. This is a difficult thing to know and more difficult to ask because it is embedded within power, conflict and potential cultural loss in AI/AN communities. A limitation in this paper is a dearth of empirical research on contemporary governance and its relationship to traditional practices. Public deliberation researchers and practitioners should inquire as to what social and cultural structures bearing on deliberation might exist. For instance, it might be worth researching whether the perspectives of young people or elders are valued differently than the rest of the population, as in the case of the Ojibwa and Yup’ik who differed along these lines of inclusion. In some contexts, those variations may be explicitly political and translate to different levels of participation, whereas in others they may be based on social and cultural norms. Another example of the differences between inclusion based on formal or constitutional practices versus social norms is that of membership and deference. If you look at tribal constitutions, any tribal member 18 or above would be equal for the purposes of contribution to a discussion, but it would be wrong to assume this in many cases, as elders or clan leaders are given greater deference in the context of deliberation. In addition, the effects of settler colonialism on AI/AN communities persist today in myriad ways that can affect deliberative engagement methods. For example, an AI/AN tribe without a land base may have less capacity for participating in deliberative discussions about land use and natural resource management.

## Conclusion

This article has several limitations. The cases we explored are not representative of all political discussion practices but rather a selected group to highlight variation. Our knowledge of traditional governing practices for this paper were informed by a limited number of sources concerning several tribal contexts rather than attempting a comprehensive understanding of a single context. Greater inclusion of alternative points of view of these governance practices would likely alter our conclusions. Alternatively, we could have worked in reverse and used Indigenous deliberative practices to reflect onto Western political systems or practices. Our intent is not to judge AI/AN governance as deficient to other governance practices, but to consider the appropriateness of employing existing conceptions of deliberation, as understood by seminal thinkers in the field such as Dahl and Gastil, in the context of AI/AN communities. Future research might turn our study on its head by drawing out AI/AN notions of valued public discourse and placing them in contrast to non-Native governing practices. Hence, such future research would follow calls to indigenize scholarship by placing AI/AN peoples and their ideas in the foreground (see Smith, 1999). Another approach future research might take is to begin to identify facilitating conditions for deliberative practices. Our argument is that we see diversity in deliberative practices rather than exploring causal relationships or associations between tribal or regional characteristics and levels or types of deliberation. Future research might identify what groups are more deliberative and link that back to subsistence, religious, or regional attributes.

Despite these limitations, our study makes some important contributions to scholarship on deliberative governance and AI/AN communities. We found several important areas of synergy between the ideals of deliberative democracy and accounts of AI/AN political discussion practices, even in examining different contexts and time periods. There are interesting variations between these different settings, which underscores a common critique that forms of community engagement must be well suited to a particular community (Lukensmeyer, 2013). It also suggests that future research should examine how deliberation and contemporary AI/AN discussion practices may overlap, conflict, and work orthogonally to each other. Rather than this paper serving as a conclusive analysis, it is intended as a starting point to this discussion, which seems vital given the rise in the use of deliberation as a practical method of engagement. Our work suggests there may indeed be some benefit in relating deliberation and AI/AN practices with each other to develop useful, robust, and appropriate methods of public engagement in contemporary AI/AN communities that are sensitive, in particular, to different understandings of authority that shape opportunities for participation and inclusion. Yet, our results suggest that deliberation must be done carefully to avoid grafting an Aristotelian worldview onto an Indigenous community with distinct values and interests, replicating the sins of colonialism.

Motivating this article is a potential discussion of the appropriateness of deliberation for Indigenous populations. As research on deliberation makes intellectual contact with Indigenous politics—either as part of Indigenous governance practices or as incorporated into deliberations hosted by non-Indigenous populations—it runs the risk of being subsumed by the question of whether principles of deliberation reinforce traditional governance or simply enact colonial power. If previous research on dialogs concerning the appropriateness of social and economic principles for AI/AN communities provides any insights, concern is certainly warranted.

### Data availability

All data analyzed during this study are previously published accounts of AIAN governance, and are cited in the reference list of the article.

## Figures and Tables

**Table 1:** Deliberative democracy according to Dahl and Gastil’s criteria in Iroquois, Yup’ik, Santa Clara Pueblo (pre and post-IRA constitution), and Ojibwa governance.

Case	Dahl			Gastil	
	
	Inclusion	Participation opportunities	Enlightened understanding	Social components of deliberation	Analytic components of deliberation
Iroquois – Great Law of Peace	Partial: - Status matters (*Hoyaneh* powerful voice) - Men at formal deliberation space	Partial: - Only *Hoyaneh* indirectly with input of women (influence of Clan Mothers over *Hoyaneh*)	Yes: - Exceeds notions of deliberation by considering those outside room and not in existence (7th Generation)	Yes: - High emphasis on respect and social position and responsibilities of speakers	Unclear: - Some components of this are described such as 7th Generation. - A broad mindedness is expected of *Hoyaneh*
Yup’ik	Partial: - Primarily male elders, but included women and young people occasionally	Yes: - Major plans for village discussed in detail with key village members	Yes: - Discussion included moral code, traditional narratives, personal experience	Yes: - Respectful discussion within the qasgi that included consideration of many views	Yes: - Options weighed before settling on plans - Discussion grounded in morals, personal experience, traditional narratives & worldview
Santa Clara Pueblo (Pre-IRA)	No: - Community not expected to participate	No: - Community not expected to participate	Yes: - Decisions in tune with culture and values, rather than individual self-interest	Not found in sources	Unclear: - Not deliberative, but emphasis is placed on underlying community values
Santa Clara Pueblo (Post-IRA)	Partial: - Voting for adult members	Partial: - Open discussion of politics, citizen influence on agenda	Unclear: - No requirement in the IRA Constitution to do so	Unclear: - No requirement in the IRA Constitution to do so	Unclear: - No requirement in the IRA Constitution to do so
Ojibwa	Partial: - Exclusion around age (young people)	Yes: - Village-level included in important decisions	Yes: - Leaders were expected to display enlightened understanding	Yes: - Expectations of respect at inter-tribal level, but not at tribal level	Partial: - Leaders expected to do this, but not necessarily individuals

## Note

1 In addition, the structure of the qasgi or qasgiq was seen as mirroring the structure of the natural world: the Yup’ik people believed marine mammals had a similar communal fire pit where they gathered to observe the world and made decisions (Fienup-Riordan, 2005).

## References

[R1] Abdel-Monem T, Bingham S, Marincic J, Tomkins A (2010) Deliberation and diversity: Perceptions of small group discussions by race and ethnicity. Small Group Res 41:746–776

[R2] Alfred T (1999) Peace, power, righteousness: an indigenous manifesto. Oxford University Press, Ontario

[R3] Ayunerak P, Alstrom D, Moses C, Charlie J, Rasmus SM (2014) Yup’ik culture and context in Southwest Alaska: community member perspectives of tradition, social change, and prevention. Am J Community Psychol 54(1–2):91–99. 10.1007/s10464-014-9652-4 24771075 PMC4119478

[R4] Barker J (ed) (2005) Sovereignty Matters: Locations of contestation and possibility in indigenous struggles for self-determination. University of Nebraska Press, Lincoln

[R5] Barsh R (1988) Contemporary Marxist theory and native American reality. Am Indian Q 12(3):187–211

[R6] Biolsi T (1998) Organizing the Lakota: the political economy of the new deal on the Pine Ridge and Rosebud Reservations. University of Arizona Press, Tucson

[R7] Britt LL, Smithberger LK (2018) Developing a civic identity by designing and facilitating public opportunities for deliberative dialogue. In: Mitchell TD, Soria KM (eds) Educating for citizenship and social justice: practices for community engagement at research universities. Palgrave Macmillan, New York, p 35–45

[R8] Burkhalter S, Gastil J, Kelshaw T (2002) A conceptual definition and theoretical model of public deliberation in small face-to-face groups. Commun Theory 12:398–422. 10.1111/j.1468-2885.2002.tb00276.x

[R9] Carcasson M, Sprain L (2016) Beyond problem solving: reconceptualizing the work of public deliberation as deliberative inquiry. Commun Theory 26(1):41–63. 10.1111/comt.12055

[R10] Carson L (2011) Designing a public conversation using the World Café method. Soc Altern 30(1):10–14

[R11] Carson L, Gastil J, Hartz-Karp J, Lubensky R (Eds) (2013) The Australian Citizens’ Parliament and the future of deliberative democracy. Penn State University Press, University Park

[R12] Carson L, Hartz-Karp J (2005) Adapting and combining deliberative designs: juries, polls, and forums. In: Gastil J, Levine P (eds) The deliberative democracy handbook: Strategies for effective civic engagement in the twenty-first century. Jossey Bass, San Francisco

[R13] Chambers S (2003) Deliberative democratic theory. Annu Rev Political Sci 6:307–326. 10.1146/annurev.polisci.6.121901.085538

[R14] Cobb AJ (2005) The national museum of the American Indian as cultural sovereignity. American Stud 57(2):484–506

[R15] Cohen J (1997) Deliberation and democratic legitimacy. In: Bohman JF, Rehg W (Eds) Deliberative democracy: essays on reason and politics. MIT Press, Cambridge

[R16] Cornell S, Gil-Swedberg M (1995) Sociohistorical factors in institutional efficacy: economic development in three American Indian cases. Econ Dev Cult Change 43(2):239–268

[R17] Cramer Walsh K (2007) Talking about race: community dialogues and the politics of difference. University of Chicago Press, Chicago

[R18] Crawford N (1994) A security regime among democracies: cooperation among Iroquois nations. Int Organ 48(3):345–385

[R19] Cronin AE, Ostergren DM (2007) Democracy, participation, and native American tribes in collaborative watershed management. Society & Natural Resources 20:527–542. 10.1080/08941920701338059

[R20] Cronon W (1983) Changes in the land: Indians, colonists, and the ecology of New England. Hill and Wang, New York

[R21] Dahl RA (1989) Democracy and its critics. Yale University Press, New Haven

[R22] Daniels SE, Walker GB (2001) Working through environmental conflict: the collaborative learning approach. Praeger, Westport

[R23] Davidson KL, Moses MS (2012) Speaking across difference in community dialogues on affirmative action policy. Equity Excell Educ 45(1):217–236. 10.1080/10665684.2012.645373

[R24] De La Fuente O (2013) Deliberative democracy vs. Politics of identity. The Age of Human Rights 1:35–48

[R25] Dozier E (1966) Hano: a Tewa Indian community in Arizona. Harcourt Brace Jovanovich, Fort Worth

[R26] Dunbar-Ortiz R (2015) An indigenous peoples’ history of the United States. Beacon Press, Boston

[R27] Eveland WP, Morey AC, Hutchens MJ (2011) Beyond deliberation: new directions for the study of informal political conversation from a communication perspective. J Commun 61:1082–1103. 10.1111/j.1460-2466.2011.01598.x

[R28] Fagotto E, Fung A (2006) Empowered participation in urban governance: the minneapolis neighborhood revitalization program. Int J Urban Reg Res 30 (3):638–655. 10.1111/j.1468-2427.2006.00685.x

[R29] Fienup-Riordan A (1994) Boundaries and passages: rule and ritual in Yup’ik Eskimo oral tradition. University of Oklahoma Press, Norman

[R30] Fienup-Riordan A (2005) Wise words of the Yup’ikPeople: we talk to you because we love you. University of Nebraska Press, Lincoln

[R31] Fienup-Riordan A (2012) Ellavut/Our Yup’ik world and weather: community and change on the Bering Sea Coast. University of Washington Press, Seattle

[R32] Fish S (2002) Islam and authoritarianism. World Politics 55:4–37

[R33] Fishkin JS (2009) When the people speak: deliberative democracy and public consultation. Oxford University Press, Oxford

[R34] Fowler L (2007) Politics. In: Biolsi T (ed) A companion to the anthropology of American Indians. p 69–94

[R35] Fung A, Wright EO (Eds) (2003) Deepening democracy: institutional innovations in empowered participatory governance. Verso, London

[R36] Gastil J (2004) Adult civic education through the National Issues Forums: Developing democratic habits and dispositions through public deliberation. Adult Educ Q 54:308–328. 10.1177/0741713604266142

[R37] Gastil J (2008) Political communication and deliberation. Sage, Thousand Oaks

[R38] Gastil J, Knobloch KR, Reedy J, Henkels M, Cramer K (2018) Assessing the electoral impact of the 2010 Oregon Citizens’ initiative review. Am Politics Res 46(3):534–563. 10.1177/1532673×17715620

[R39] Gastil J, Levine P (Eds) (2005) The deliberative democracy handbook: strategies for effective civic engagement in the twenty-first century. Jossey Bass, San Francisco

[R40] Goi S (2005) Agonism, deliberation, and the politics of abortion. Polity 37:54–81. 10.1057/palgrave.polity.2300005

[R41] Graham L (2008) Reparations, self-determination and the seventh generation. Harv Hum Rights J 21:47; Suffolk University Law School Research Paper No. 07-22. Available at SSRN: https://ssrn.com/abstract=1126774

[R42] Habermas J (1996) Between facts and norms: Contributions to a discourse theory of law and democracy. MIT Press, Cambridge

[R43] Harris A, Johnson T (Eds) (2009) Haudenosaunee guide for educators. National Museum of the American Indian, Smithsonian Institution Washington, Smithsonian Institute. https://americanindian.si.edu/sites/1/files/pdf/education/HaudenosauneeGuide.pdf. Accessed 6 2019

[R44] Hensel C (1996) Telling our selves: ethnicity and discourse in Southwestern Alaska. Oxford University Press, Oxford

[R45] Hickerson A, Gastil J (2008) Assessing the difference critique of deliberation: gender, emotion, and the jury experience. Commun Theory 18(2):281–303

[R46] Hill W, Lange C (1982) An ethnography of Santa Clara Pueblo, New Mexico. University of New Mexico Press, Albuquerque

[R47] Horn-Miller K (2013) What does indigenous participatory democracy look like? Kahnawà:Ke’s community decision making process. Rev Const Stud 18(1):5

[R48] Ivison D (2010) Deliberative democracy and the politics of reconciliation. In Kahane D, Weinstock D, Leydet D, & Williams M (eds) Deliberative democracy in practice. UBC Press, Vancouver, p 115–137

[R49] Jacobs R (1991) The Iroquois Great Law of Peace and the United States Constitution: how the founding fathers ignored the Clan mothers. Am Indian Law Rev 16(2):497–531

[R50] Jacobs LR, Cook FL, Delli Carpini MX (2009) Talking together: public deliberation and political participation in America. University of Chicago Press, Chicago

[R51] Jacobson AW. Alaska Native Language Center. 1998. Yup’ik stories read aloud=Yugcetun Qulirat Naaqumalriit Erinairissuutmun. Alaska Native Language Center, Fairbanks

[R52] Johansen B (1982) Forgotten Founders: how the American Indian helped shape democracy. Harvard Common Press, Cambridge

[R53] Judkins B (2012) Intergroup dialogues, building community and relational justice. Catalyst: Soc Justice Forum 2(1). http://trace.tennessee.edu/catalyst/vol2/iss1/6

[R54] Karpowitz CF, Raphael C (2014) Deliberation, democracy, and civic forums. 10.1017/CBO9781107110212

[R55] Karpowitz CF, Raphael C, Hammond AS IV (2009) Deliberative democracy and inequality: two cheers for enclave deliberation among the disempowered. Politics Soc 37:576–615. 10.1177/0032329209349226

[R56] Kelly LC (1983) The assault on assimilation: John Collier and the origins of Indian policy reform. University of New Mexico Press, Albuquerque

[R57] Knobloch KR (2011) Public sphere alienation: a model for analysis and critique. Javnost—Public 18(4):21–37. 10.1080/13183222.2011.11009065

[R58] Knobloch KR, Gastil J (2015) Civic (re)socialisation: the educative effects of deliberative participation. Politics 35(2):183–200. 10.1111/1467-9256.12069

[R59] Knobloch KR, Gastil J, Reedy J, Cramer Walsh K (2013) Did they deliberate? Applying an evaluative model of democratic deliberation to the Oregon Citizens’ Initiative Review. J Appl Commun Res 41:105–125. 10.1080/00909882.2012.760746

[R60] Krech S (1999) The ecological Indian: myth and history. W. W. Norton & Company, New York

[R61] Lansing S (2007) Priests and Programmers: Technologies of Power in the Engineered Landscape of Bali. Princeton University Press, Princeton, NJ

[R62] Latimore J, Nolan D, Simons M, Khan E (2017) Reassembling the Indigenous Public Sphere. Australasian Journal of Information Systems 21

[R63] Lemont ED (2006) American Indian Constitutional reform and the rebuilding of native nations. University of Texas Press, Austin

[R64] Lerma M (2012) Indigeneity and homeland: land, history, ceremony, and language. Am Indian Cult Res J 36(3):75–98

[R65] Lerma M (2014) Indigenous sovereignty in the 21st century: knowledge for the indigenous spring. Florida Academic Press, Gainesville

[R66] Lijphart A (1971) Comparative politics and comparative method. Am Political Sci Rev 65:682–693

[R67] Locke J (1690) An essay concerning human understanding

[R68] Lukensmeyer C (2013) Bringing citizen voices to the table. Jossey Bass, San Francisco

[R69] Lundmark C, Matti S (2015) Exploring the prospects for deliberative practices as a conflict-reducing and legitimacy-enhancing tool: the case of Swedish carnivore management. Wildlife Biol 21(3):147–156

[R70] Mac Ginty R (2008) Indigenous peace-making versus the liberal peace. Cooperation Confl 43(2):139–163

[R71] Makariev P (2013) Cultural Rights and Deliberative Policy: Beyond Habermas’ “Between Facts and Norms.” Journal of Public Deliberation 9:2

[R72] Mansbridge J, Hartz-Karp J, Amengual M, Gastil J (2006) Norms of deliberation: an inductive study. J Public Deliberation 2(1). http://www.publicdeliberation.net/jpd/vol2/iss1/art7

[R73] Marques ÂCS, Maia RCM (2010) Everyday conversation in the deliberative process: An analysis of communicative exchanges in discussion groups and their contributions to civic and political socialization. J Commun 60:611–635. 10.1111/j.1460-2466.2010.01506.x

[R74] Mendelberg T, Oleske J (2000) Race and public deliberation. Political Commun 17:169–191

[R75] Mill JS (1859) On liberty. Penguin Books, London

[R76] Miller C (2010) Ogimaag: Anishinaabeg leadership, 1760–1845. University of Nebraska Press, Lincoln

[R77] Morgan LH (1901) League of the Ho-de-no-sau-nee or Iroquois.

[R78] Dodd, Mead Mohawk J (1978) A basic call to consciousness. Awkwesasne Notes, Rooseveltown

[R79] Molina-Markham E (2014) Finding the “Sense of the Meeting”: Decision making through silence among quakers. West J Commun 78:155–174

[R80] Nabatchi T, Gastil J, Leighninger M, Weiksner GM (Eds) (2013) Democracy in motion: evaluating the practice and impact of deliberative civic engagement. Oxford University Press, New York

[R81] Nadasdy P (2005) Transcending the debate over the ecologically Noble Indian: indigenous peoples and environmentalism. Ethnohistory 52(2):291–331

[R82] Nagel J (1996) American Indian ethnic renewal: red power and resurgence of identity and culture. Oxford University Press, Oxford

[R83] Napoleon H, Madsen EC, Alaska Native Knowledge Network (1996) Yuuyaraq: the way of the human being. Alaska Native Knowledge Network, University of Alaska, Fairbanks, Fairbanks

[R84] Norcini M (2005) The political process of factionalism and self-governance at Santa Clara Pueblo, New Mexico. Proc Am Philos Soc 149(4):544–590

[R85] O’Doherty KC, Burgess MM (2009) Engaging the public on biobanks: outcomes of the BC Biobank Deliberation. Public Health Genomics 12(4):203–215. 10.1159/000167801 19367089

[R86] Orr R (2017) Reservation politics: historical trauma, economic development and intratribal conflict. The University of Oklahoma Press, Norman

[R87] Orr R, Ruppanner L (2016) ‘Indian time’ for nature? A multi-level approach to American Indian outdoor time in everyday life Ethn Racial Stud 39 (7):1223–1241

[R88] Ortiz A (1969) The Tewa world; space, time, being, and becoming in a Pueblo Society. University of Chicago Press, Chicago

[R89] Pinel SL (2007) Culture and Cash: How Two New Mexico Pueblos Combined Culture and Development. Alternatives: Global, Local, Political 32

[R90] Rasmus SM, Trickett E, Charles B, John S, Allen J (2019) The Qasgiq model as an indigenous intervention: using the cultural logic of contexts to build protective factors for Alaska Native suicide and alcohol misuse prevention. Cult Divers Ethn Minor Psychol 25(1):44–54. 10.1037/cdp0000243. PMC6563829 30714766

[R91] Ratner RS (2005) The B.C. Citizen’s Assembly: the public hearings and deliberations stage. Can Parliam Rev 28(1):24–33

[R92] Rawls J (2005) Political liberalism. Columbia University Press, New York

[R93] Raymond-Yakoubian J, Daniel R (2018) An Indigenous approach to ocean planning and policy in the Bering Strait region of Alaska. Mar Policy 97:101–108. 10.1016/j.marpol.2018.08.028

[R94] Reedy J, Gastil J, Moy P (2016) From the secret ballot to the public vote: examining voters’ experience of political discussion in vote-by-mail elections. Political Commun 33(1):39–58. 10.1080/10584609.2014.969462

[R95] Reo N, Whyte K (2011) Hunting and morality as elements of traditional ecological knowledge. Hum Ecol 40(1):15–27

[R96] Richards RC, Gastil J (2015) Symbolic-cognitive proceduralism: a model of deliberative legitimacy. J Public Deliberation 11(2). http://www.publicdeliberation.net/jpd/vol11/iss2/art3

[R97] Rihoux B, Ragin C (2003) Configurational comparative methods: qualitative comparative analysis (QCA) and related techniques. Sage, Thousand Oaks

[R98] Rockloff SF, Lockie S (2006) Democratization of coastal zone decision making for Indigenous Australians: Insights from stakeholder analysis. Coast Manage 34 (3):251–266

[R99] Ryfe DM (2006) Narrative and deliberation in small group forums. J Appl Commun Res 34(1):72–93. 10.1080/00909880500420226

[R100] Sanders LM (1997) Against deliberation. Political Theory 25:347–376. 10.1177/0090591797025003002

[R101] Sayre GM (2000) Les Sauvages Américains: Representations of Native Americans in French and English Colonial Literature. University of North Carolina Press, Chapel Hill

[R102] Simpson A (2014) Mohawk Interruptus: political life across the borders of Settler States. Duke University Press, Durham

[R103] Siu A, Stanisevski D (2013) Deliberation in multicultural societies: addressing inequality, exclusion, and marginalization. In: Nabatchi T, Gastil J, Leighninger M, Weiksner GM (Eds) Democracy in motion: evaluating the practice and impact of deliberative civic engagement. Oxford University Press, Oxford

[R104] Smith LT (1999) Decolonizing methodologies: research and indigenous peoples. Zed Books, London

[R105] Souza C (2001) Participatory budgeting in Brazilian cities: limits and possibilities in building democratic institutions. Environ Urban 13(1):159–184. 10.1177/095624780101300112

[R106] Strickland CJ, Squeoch MD, Chrisman NJ (1999) Health promotion in cervical cancer prevention among the Yakama Indian women of the Wa’Shat Long-house. J Transcult Nurs 10(3):190–196 10693405 10.1177/104365969901000309

[R107] Sturtevant WC, Trigger BG, Smithsonian Institution (1978) Handbook of North American Indians: vol 15. Smithsonian Institution, Washington

[R108] Tocqueville AD (1835) Democracy in America. Library of America, New York (trans: Goldhammer A)

[R109] Tooker E (1978) The league of the Iroquois: its history, politics, and ritual. Handb North Am Indians 15:418–441

[R110] Ugarriza JE, Caluwaerts D (Eds) (2014) Democratic deliberation in deeply divided societies: from conflict to common ground. Palgrave Macmillan, London

[R111] Valadez JM (2010) Deliberation, cultural difference, and indigenous self-governance. Good Soc 19(2):60–65

[R112] von der Porten S, de Loë RC (2013) Collaborative approaches to governance for water and Indigenous peoples: A case study from British Columbia, Canada. Geoforum 50:149–160

[R113] Von der Porten S, De Loë R, Plummer R (2015) Collaborative environmental governance and indigenous peoples: recommendations for practice. Environ Pract 17(2):134–144. 10.1017/S146604661500006X

[R114] Walmsley H (2010) Biobanking, public consultation, and the discursive logics of deliberation: Five lessons from British Columbia. Public Underst Sci 19 (4):452–468 20977183 10.1177/0963662509335523

[R115] Weaver SM (1984) Seth Newhouse and the Grand River Confederacy at mid-nineteenth century. In: Foster Michael K, Campisi Jack, Mithun Marianne (eds) Extending the rafters: interdisciplinary approaches to Iroquoian studies. State University of New York Press, Albany NY, p 165–182

[R116] Wheatley S (2003) European Journal of International Law. Volume 14, Issue 3, p 507–527

[R117] White R (1995) The Organic Machine: The Remaking of the Columbia River Richard White. Hill and Wang, New York

[R118] Williams KP (2018) Kayanerenkó:wa: the Great Law of peace. University of Manitoba Press, Winnipeg, Manitoba

[R119] Wojcieszak ME, Mutz DC (2009) Online groups and political discourse: do online discussion spaces facilitate exposure to political disagreement? J Commun 59 (1):40–56. 10.1111/j.1460-2466.2008.01403.x

[R120] Young IM (2002) Inclusion and democracy. Oxford University Press, Oxford

